# Evaluation of Lower Dental Arch Crowding and Dimension after Treatment with Lip Bumper versus Schwarz Appliance. A Prospective Pilot Study

**DOI:** 10.3390/dj8020034

**Published:** 2020-04-10

**Authors:** Vincenzo Quinzi, Silvia Caruso, Stefano Mummolo, Alessandro Nota, Anna Maria Angelone, Antonella Mattei, Roberto Gatto, Giuseppe Marzo

**Affiliations:** 1 Department of Health, Life and Environmental Science, University of L’Aquila, Piazza Salvatore Tommasi, 67100 L’Aquila, Italy; vincenzo.quinzi@univaq.it (V.Q.); silvia.caruso@univaq.it (S.C.); annamaria.angelone@univaq.it (A.M.A.); antonella.mattei@cc.univaq.it (A.M.); roberto.gatto@univaq.it (R.G.); giuseppe.marzo@univaq.it (G.M.); 2 Dental School, Vita-Salute San Raffaele and IRCCS San Raffaele Hospital, 20132 Milan, Italy; nota.alessandro@hsr.it

**Keywords:** mixed dentitions, interceptive orthodontics, preventive orthodontics, corrective orthodontics, dental arch, mandible

## Abstract

**Aim:** The treatment of patients with mixed dentition, with inferior moderate dental crowding (the so-called borderline cases, between extraction and expansion) is not yet clear. Two examples of widely used appliances for increasing lower dental arch dimensions are the Schwarz’s appliance and lip bumper. The aim of this prospective study was to compare dental crowding and arch dimensions from pre- to post-treatment with lip bumper versus Schwarz’s appliance. **Subjects and Methods:** Pre- and post-treatment orthodontic records of twenty subjects (10 males and 10 females) were analyzed in the present study. Inclusion criteria were: first/second molar class malocclusion; crowding of the mandibular arch, from mild to moderate (4–6 mm); mixed dentition; age ≤ 9 years at the beginning of the treatment; stage CS1 or CS2 of maturation of the cervical vertebrae analysis (CVM) at the beginning of the treatment. Ten subjects were treated with a lip bumper, and ten with the removable Schwarz appliance. The primary outcomes were the variations in dental crowding and arch dimensions from pre- to post-treatment. **Results:** Both the two appliances caused a statistically significant mean improvement/reduction in crowding, of 3.5 mm and 2.9 mm, for the Schwarz appliance and lip bumper, respectively. The Schwarz appliance resulted more effective in increasing arch dimension at the intercanine level, and arch perimeter, while the lip bumper achieves a higher increase in arch length. **Conclusions:** A lip bumper and Schwarz appliance are both useful in reducing crowding in mixed dentition. This improvement is due to the increase in dental arch dimensions, although the distribution of space resulted slightly differently between the two appliances.

## 1. Introduction

Crowding is the most frequent form of malocclusion, as stated in the United States at the turn of the 1990s, when a study on the prevalence of dental malocclusions was conducted in a sample of 14,000 subjects representing the American population in the context of the N.H.A.N.E.S. III (National Health and Nutrition Estimates Survey) [1]. In that survey, the prevalence of crowding was observed in about 45% of children in mixed dentition, and in 66.5% of subjects aged from 18 to 50 years. A similar prevalence of crowding in mixed dentition was also reported in the Italian population [2,3,4]. Therefore, crowding is considered a malocclusion that never self-corrects, as rather it worsens over time. If present in deciduous teeth, it will worsen in the next two dental stages [5].

In general, during the years following the completion of dentition [6], it worsens and involves 50% of individuals who were exempt from it during their first decade of life [1]. The worsening of the crowding seems to occur concomitantly with the physiological decrease in the length of the dental arches, and is associated with aesthetic as well as periodontal problems [7,8], mostly in adults [9] and with other malocclusions, such as crossbite [10,11], class II malocclusion [12,13], or temporomandibular joint dysfunction [11,14,15].

In the past 100 years, the debate between the two treatments of crowding—extracting teeth or dental arches expansion—has not yet seen a universal winner among modern orthodontists.

In patients with slight or severe dento-alveolar discrepancies, the choice between extracting teeth or gaining space with suitable techniques, such as the use of mini-screw anchorage [16], is clear [17].

On the other hand, the path to follow for those patients with moderate dental crowding, the so-called borderline cases, is less clear.

The “extraction or non-extraction” controversy arises from when Edward Angle opposed the extraction treatment because of a purely anatomical observation, that was that the lower jaw does not have a violable suture line. Consequently, if in the upper arch it was therefore possible to increase the transverse diameters by separating the mid-palatine suture, this was not practicable in the lower arch. So, although rapid palatal expansion has shown to be able to induce significant changes also in the mandibular arch, through a change in the tongue position [18,19,20,21], which tends to improve its diameter—often compressed in the contracted maxilla—this is not always sufficient to resolve larger crowdings [22].

A space-saving procedure in the lower arch in patients with mixed dentition is possible with devices that induce a remodeling of the alveolar bone, and an improvement of teeth inclination in the posterior sectors, where they are often too lingualized. This procedure usually translates into an increase in arch perimeters and transverse dimensions of the lower dental arch.

One of the most used appliances is the lip bumper (LB), which alone can obtain flaring of the mandibular incisors, distalization and uprighting of the mandibular first molars, and buccal expansion of the canines, premolars, and molar transverse diameters. Lip forces are transmitted through this appliance onto the molars [23]. It has been stated that intermolar and interpremolar changes with a LB during mixed dentition can be considered as the best predictors for post-retention mandibular dental arch stability, as for every millimeter of increase in the intermolar and interpremolar widths there was a 1.52 and 2.70 times increase, respectively, in the odds of having stability. There was also weak evidence for the influence of gender [24]. Another appliance used is the Schwarz appliance (SA), a removable plate that primarily affects the dento-alveolar complex, while it has little effect on either mandibular bodies [25]. The SA is also used for the expansion of the upper arch [26].

The purpose of this clinical trial was to evaluate the dimensional variations of the mandibular arches in patients with mixed dentition affected by mild to moderate dental crowding, treated with two different types of orthodontic devices—the LB and the SA.

## 2. Materials and Methods

The present protocol was approved by the Ethics Committee of the University of L’Aquila (Document DR206/2013, 16 July 2013). Written consent was obtained from all the participants (patient’s parents/legal guardians). Twenty patients (10 males and 10 females), referred to the Department of Orthodontics, University of L’Aquila (Italy) for orthodontic needs, were enrolled in the present study.

**Figure 1:**
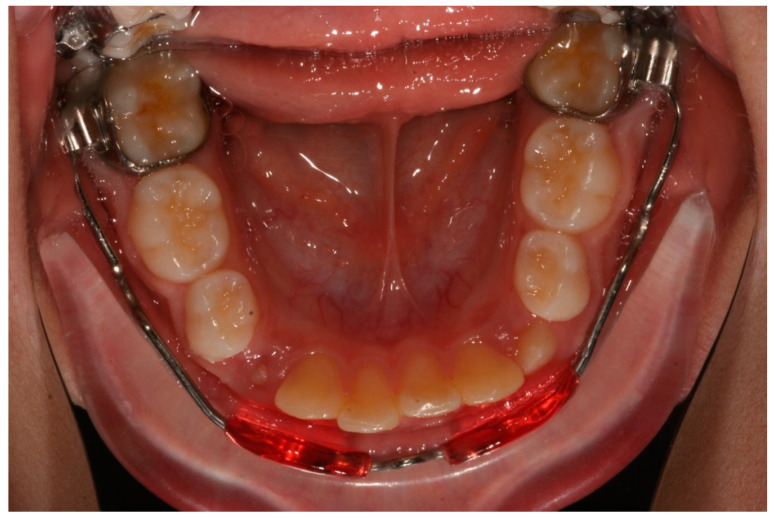
Lip bumper appliance.

**Figure 2:**
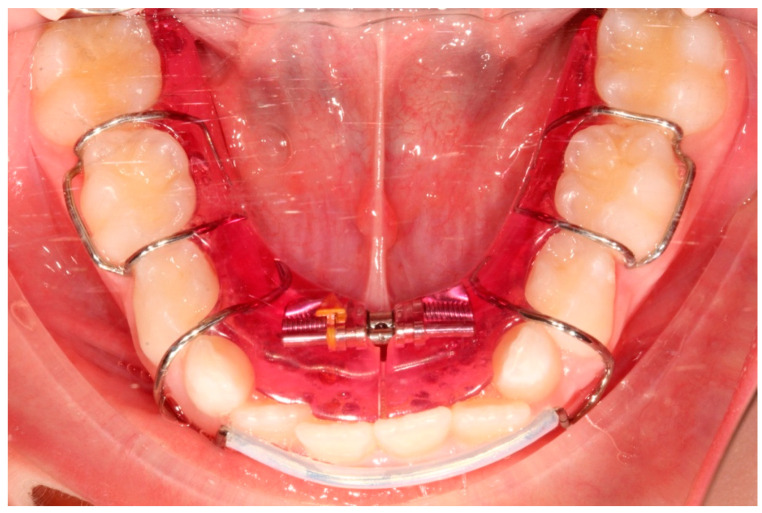
Schwarz appliance.

Ten subjects were treated with LB (Figure 1), and ten with the removable SA (Figure 2).

The assignment to either study group took place by simple randomization with sealed envelopes. The inclusion criteria for this study were: first/second molar class malocclusion, mild to moderate crowding of the mandibular arch (4–6 mm), mixed dentition, age ≤ 9 years at the beginning of the treatment, and stage CS1 or CS2 of maturation of the cervical vertebrae analysis (CVM) at the beginning of the treatment.

Patients who underwent previous orthodontic treatments, presented craniofacial anomalies, or underwent teeth extraction treatments were excluded.

Measurements were taken on the plaster models before and after treatment with a digital caliper, following the same protocol reported in a previous article by Raucci et al. [24].

**Figure 3:**
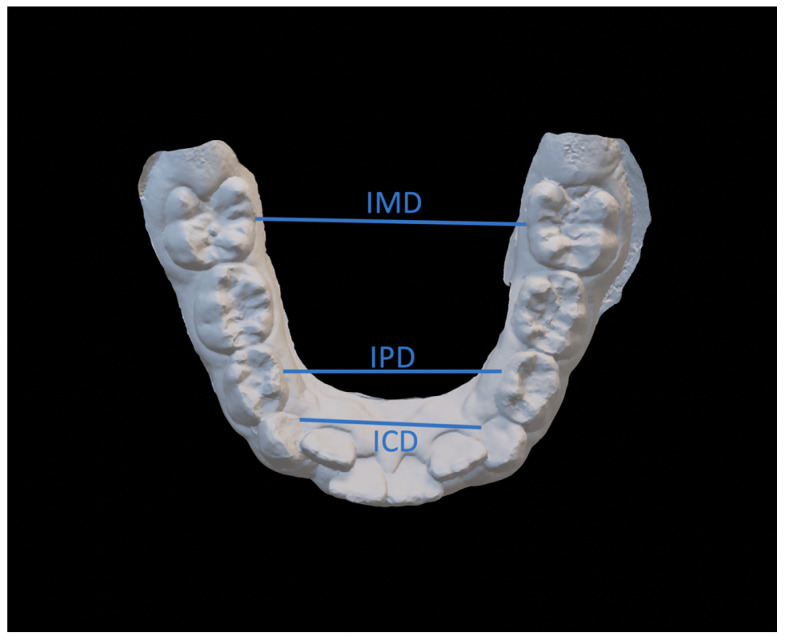
Intercanine distance (ICD), interpremolar distance (IPD), intermolar distance (IMD).

**Figure 4:**
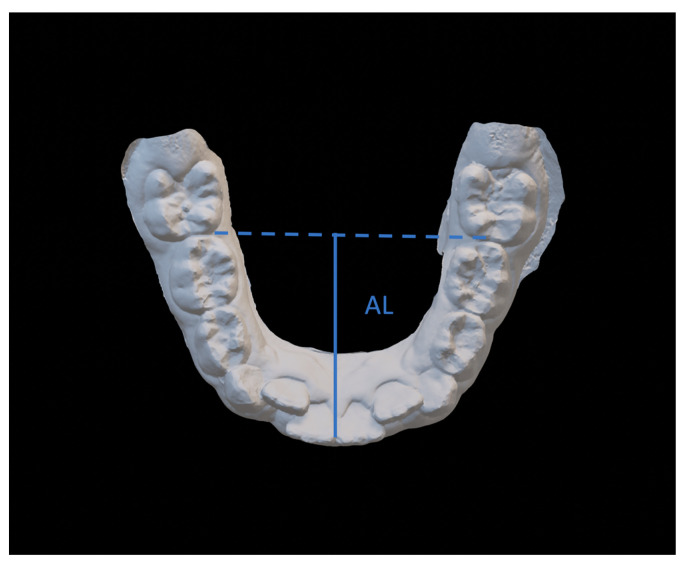
Arch length (AL).

**Figure 5:**
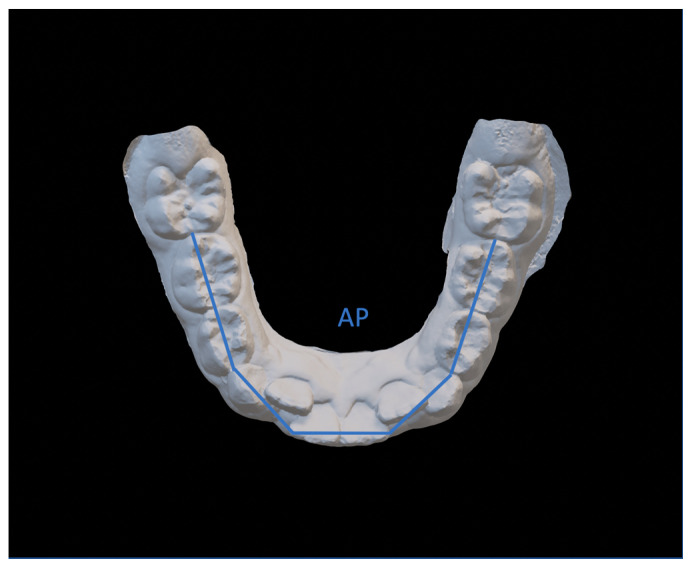
Arch perimeter (AP).

The variables were (Figure 3, Figure 4 and Figure 5):

- Intercanine distance (ICD), calculated as the distance between the innermost lingual point of the gingival margin of the deciduous or permanent canines;
- Interpremolar distance (IPD), calculated as the distance between the innermost lingual point of the gingival margin of the first bicuspids or first deciduous molars;
- Intermolar distance (IMD), calculated as the distance between the points of intersection of the lingual sulcus with the cervical gingival margin of the first permanent molars;
- Arch length (AL), measured as the perpendicular distance between the most vestibular point between the lower central incisors, and the connection line between the mesial contact points of the first permanent molars;
- Arch perimeter (AP), calculated as the sum of the distances between: mesial contact point of the first permanent molar; mesial contact point of the first bicuspid; mesial contact point of the first bicuspid; point of mesial lateral incisive contact; point of lateral mesial incisor contact; contralateral central incisor distal contact point.
- Crowding (CRO), evaluated as the tooth-size/arch length discrepancy.

### Statistical Analysis

A descriptive analysis of the investigated variables was conducted using frequency and percentage for the nominal variables and mean and standard deviation for the continuous variables. The differences in age and gender between children treated with SA and LB were assessed using the χ^2^ test and the Mann–Whitney test. A two-way ANOVA for repeated measurements was performed to assess the statistical significance of the differences between the averages of the measurements and the two treatments in the two observation times. The tests used are bidirectional and used with a significance level of 5%. The data were processed using the statistical software Stata: 15/IC.

## 3. Results

Table 1 shows the distribution of the sample by gender and age, and their clinical characteristics before treatment (t0). The two groups were homogeneous in measurements pre-treatment and in age and gender distribution. There was a statistically significant difference between the duration of the two different treatments, that was 18.8 months on average for the SA, and 11.7 months for the LB.

Table 2 shows the distribution of the variables before and after the orthodontic treatment. Both the two appliances caused a statistically significant improvement/reduction in crowding, of 3.5 mm and 2.9 mm, for SA and LB, respectively.

While with both appliances the crowding reduction was related with an increase in the dental arch dimension, statistically significant differences were observed in comparisons of the mean variations between the two groups.

ICD showed an average statistically significant increase of 3.2 mm with SA, while LB registered no significant increase.

IPD and IMD showed statistically significant increases, both with the two appliances; but those changes were higher with SA compared with LB (4.4 mm and 1.1 mm respectively).

A statistically significant increase of 9.4 mm of AL following treatment with LB was observed, while it remained almost unchanged with the SA. On the contrary, AP increased on average by 3.6 mm with SA, while it slightly decreased with the LB.

## 4. Discussion

The present study evaluated the effects of two lower arch expansion devices in reducing mandibular crowding by increasing the lower arch dimensions.

The SA allowed a statistically significant increase in ICD (*p* < 0.001), IPD (*p* < 0.001), IMD (*p* < 0.001), APD (*p* < 0.001) and a reduction in CRO (*p* < 0.001), while the LB treatment was associated with a significant increase in IPD (*p* = 0.046), IMD (*p* = 0.027), ALD (*p* < 0.001), APD (*p* < 0.001) and a decrease in CRO (*p* < 0.001) parameters. Both the devices seem to be effective in significantly reducing crowding (∆ CRO = *p* < 0.001).

In a previous study, a crowding reduction of 3.2 mm was observed with LB [24,27], while in the present study, there was a crowding reduction of 2.9 mm. In addition, that study stated an increase in intercanine, interpremolar, and intermolar widths of 3.8, 3.3, and 3.9 mm, respectively, while in the present study, changes of 0.5 mm, 1.1 mm and 1.1 mm were recorded. The difference between the two studies can be related to the considerably different treatment durations, as in the present sample, the LB was used for about 11.7 months, while in that study it was used for about two years. The present data confirm that mandibular dental arch dimensions are significantly changed after LB treatment. All these changes generate a decrease in crowding that can be clinically relevant. In the present study, no data about follow-up were recorded.

While in the present study a significant decrease in crowding was observed after treatment with SA, there are no previous data in the literature about the changes of lower dental arch dimensions after a treatment with this appliance. Looking at the present results, SA seems to be more effective with respect to the LB in achieving a higher increase in the intercanine dimension, with possible clinical implications in the prevention of the lower canine impaction [28,29].

Differently, SA’s effect on mandibular arch length is reduced if compared with the LB; this difference between the two appliances can be related to their different biomechanics, as the LB works mainly on a longitudinal plane by molar distalization and anterior arch development, while the SA works mainly with transversal expansive forces on canines, deciduous molars (premolars) and permanent first molars. The LB works on dental arch expansion indirectly, through the tongue eccentric forces [30], due to the reduction in impact of the lip concentric force. Both of the two appliances are removable and widely used in orthodontic clinics as they can guarantee a good control of oral hygiene, different from a fixed orthodontic device [31]. In the scientific literature, treatments with the SA in mixed dentition have been associated with an increase in eruption complications of the mandibular second molar [32]. For that reason, it is recommended to clinicians to monitor these patients carefully to prevent impaction of the second molars.

The differences between the variations of the lower arch’s dimensions induced by SA and the LB could be important in the clinical choice of the ideal appliance for each subject, according to arch shape and peculiar condition, such as, for example, the case of a recent temporo-mandibular joint dysfunction, that could require a treatment with a splint while expanding the dental arch (indication for SA) [14].

In addition, the SA is a removable appliance that could be better cleaned, instead of the fixed bands anchored to teeth necessary for the LB treatment [33].

As a higher intercanine increase was observed in the SA group, it could be interesting to encourage future studies about the influence of these appliances on the risk of mandibular canine impaction. The present study has also the limitation of reduced sample size that should be overcome by future studies.

## 5. Conclusions

The present study showed that the lip bumper and the Schwarz appliance are both able to reduce crowding when used in prepubertal subjects with mixed dentition, achieving an increase in dental arch perimeter, and transverse diameters, albeit with slight differences between the two devices, in the distribution of space, caused by the different mechanisms of actions of the two appliances.

## Figures and Tables

**Table 1:** Demographic and clinical characteristics before treatment.

	Whole Sample (*n* = 20)	SA (*n* = 10)	LB (*n* = 10)	*p* Value
**Gender**, n (%) ^a^				
Males	10 (50%)	5 (50%)	5 (50%)	ns
Females	10 (50%)	5 (50%)	5 (50%)	
**Age**				
(Mean ± SD) ^b^	8.46 ± 0.45	8.5 ± 0.53	8.43 ± 0.38	ns
Treatment duration				
(Mean ± SD) ^b^	15.25 ± 5.18	18.8 ± 4.52	11.7 ± 2.87	0.0014 *
Molar class, n (%) ^a^				
Molar class I	10 (50%)	5 (50%)	5 (50%)	ns
Molar class II	10 (50%)	5 (50%)	5 (50%)	

^a^ χ^2^ test. ^b^ Mann-Whitney test. ns: no statistically significant difference between groups. *: statistically significant difference between groups.

**Table 2:** Distribution of data before and after the orthodontic treatment, with statistically significant differences, and the interaction between time and treatment.

	SA (cm) (Mean ± SD)		SA (Pre vs. Post) *p*-Value	LB (cm) (Mean ± SD)		LB (Pre vs Post) *p*-Value	Interaction Time#Treatment (*p*-Value)
	Pre	Post		Pre	Post		
**ICD**	2.01 ± 0.14	2.33 ± 0.19	(0.32) < 0.001 *	2.19 ± 0.01	2.24 ± 0.12	(0.05) 0.171	<0.001
**IPD**	2.36 ± 0.1	2.8 ± 0.24	(0.44) < 0.001 *	2.57 ± 0.11	2.68 ± 0.12	(0.11) 0.046 *	0.002
**IMD**	3.14 ± 0.16	3.58 ± 0.23	(0.44) < 0.001 *	3.26± 0.11	3.37 ± 0.07	(0.11) 0.027 *	0.001
**AL**	2.31 ± 0.19	2.26 ± 0.23	(−0.05) 0.057	2.42 ± 0.09	3.36 ± 0.05	(0.94) < 0.001 *	<0.001
**AP**	7.25 ± 0.42	7.61 ± 0.46	(0.36) < 0.001 *	7.54 ± 0.22	7.2 ± 0.21	(−0.34) < 0.001 *	<0.001
**CRO** **(mm)**	−5.0 ± 2.1	−2.5 ± 2.4	(3.5) < 0.001 *	−4.6 ± 1.2	−1.7 ± 1.2	(2.9) < 0.001 *	0.351

* Two-way ANOVA for repeated measures significant differences (*p* < 0.05). Interaction time#treatment; significance indicates that the treatments show different results over time.
